# Molecular Biology Can Change the Classic Laboratory Approach for Intestinal Protozoan Infections

**DOI:** 10.3389/fmicb.2017.02191

**Published:** 2017-11-07

**Authors:** Fabio Formenti, Matteo Valerio, Massimo Guerriero, Francesca Perandin, Barbara Pajola, Manuela Mistretta, Stefano Tais, Monica Degani, Zeno Bisoffi

**Affiliations:** ^1^Centre for Tropical Diseases, Sacro Cuore Don Calabria Hospital, Verona, Italy; ^2^Medical Oncology, Sacro Cuore Don Calabria Hospital, Verona, Italy; ^3^Department of Computer Science, University of Verona, Verona, Italy

**Keywords:** real-time PCR, protozoa, diagnosis, molecular biology, laboratory

## Abstract

For many years microscopy has been considered the mainstay of the diagnosis of parasitic infections. In our laboratory, before the advent of molecular biology, the approach for the identification of parasitic infections in stools was the microscopic exam of three samples. Once we adopted molecular biology, a real-time PCR on one single sample was added to the classical coproparasitological exam of three samples. Given the high sensitivity of real-time PCR (Rt-PCR), we then decided to evaluate if a change of our routine was justified. In detail, we intended to assess if a much more practical routine, based on the analysis of a single fecal sample, was sufficiently sensitive to replace the routine described above. The new approach to be evaluated included, on the same and unique fecal sample, a classical coproparasitological exam plus Rt-PCR. The data obtained showed that the sensitivity of the new proposed approach remains very high, despite the reduction of coproparasitological exams from three to one, with the advantage of reducing costs and saving time, both for patients and for the laboratory.

## Introduction

Microscopy is the classical procedure for diagnosing parasitic infections, including the identification of protozoan trophozoites and cysts in feces, and is still the primary, often only, test offered by most routine diagnostic services.

Because several intestinal parasites are shed intermittently, patients are usually asked to deliver multiple stool samples for examination (Danciger and Lopez, 1975; Cartwright, 1999). Although this procedure is relatively simple, and allows for the identification of both helminth eggs and larvae, as well as protozoan cysts and vegetative forms, microscopy clearly has its limitations and is a time consuming procedure (Utzinger et al., 2010). Some species are difficult or even impossible to differentiate (e.g., the complex *Entamoeba histolytica-E. dispar-E. moshkovskii*), moreover the identification of any parasite highly depends on the skills and accuracy of the microscopist (Visser et al., 2006). Many laboratories perform fecal concentration techniques and/or staining to improve the diagnostic yield, thus further increasing the workload.

Alternative approaches have been developed to improve the diagnosis of enteric parasitic infections, and recently molecular approaches have been described, in particular gene amplification methods (Bretagne and Costa, 2006; Murray and Cappello, 2008). In the last decades, real-time PCR (Rt-PCR) has become widely used in many laboratories. This technique allows highly sensitive and specific identification of parasite DNA. With the use of different fluorescent labels, multiple targets can be identified simultaneously within a single reaction tube (Multiplex Rt-PCR) (ten Hove et al., 2007). Many studies have shown good accuracy of Rt- PCR on parasites such as *Giardia duodenalis* (Verweij et al., 2003b), *E. histolytica, E. dispar* (Verweij et al., 2000)*, Blastocystis* sp. (Stensvold et al., 2012), *Dientamoeba fragilis* (Verweij et al., 2007), *Strongyloides stercoralis* (Rocca et al., 2016)*, Schistosoma* sp. (ten Hove et al., 2008), and many other. A number of parasitology reference laboratories have introduced, in addition (or as an alternative) to stool microscopy (multiplex) Rt-PCR as their frontline test for the diagnosis of enteric protozoa, following the trend previously observed in clinical virology and bacteriology (Amar et al., 2007; Gunson et al., 2008; Liu, 2008; Bruijnesteijn van Coppenraet et al., 2009; Muldrew, 2009; Weile and Knabbe, 2009).

In our laboratory, before the advent of molecular biology, the approach for the identification of a parasitic infections in stools was the microscopic exam of three samples, collected on alternate days and submitted to formol-ether concentration (Buonomini et al., 1956). The collection of three samples on alternate days was laborious for the patients and time consuming for the laboratory as well. Once we adopted molecular biology, a real time PCR on one single sample was added to the classical coproparasitological exam of three samples. This new routine was adopted in January, 2014 and basically comprised two multiplex Rt-PCR for protozoa (*E. histolytica/E. dispar/Cryptosporidium* sp., and *D. fragilis/Giardia duodenalis/Blastocystis* sp.), developed according to the literature (Verweij et al., 2000, 2003b, 2007; Jothikumar et al., 2008; Stensvold et al., 2012) and substantially increasing the diagnostic yield for the targeted protozoa. Based on epidemiological considerations, a few multiplex Rt-PCR for helminths were also used in selected cases. Given the high sensitivity of Rt-PCR, we then decided to evaluate if a change of our routine was justified. In detail, we intended to assess if a much more practical routine, based on the analysis of a single fecal sample, was sufficiently sensitive to replace the routine described above. The new approach to be evaluated included, on the same and unique fecal sample, a classical coproparasitological exam plus Rt-PCR. In the present study, we compare (for intestinal protozoan infections) this new diagnostic approach with the previous routine based on the coproparasitological exam of three samples plus Rt-PCR on one sample.

## Materials and Methods

### Study Design

This observational retrospective study was conducted on the clinical records of all the patients of the Centre for Tropical Diseases (CTD), Negrar, Verona, Italy, who were requested stool microcopy and Rt-PCR for intestinal protozoa between 01.01.2014 and 31.12.2015.

### Ethics Statement

The study protocol received ethical clearance by the local competent Ethics Committee (Comitato Etico per la Sperimentazione Clinica delle Province di Verona e Rovigo, protocol number 18526).

### Participants

#### Eligibility Criteria

**FIGURE 1:**
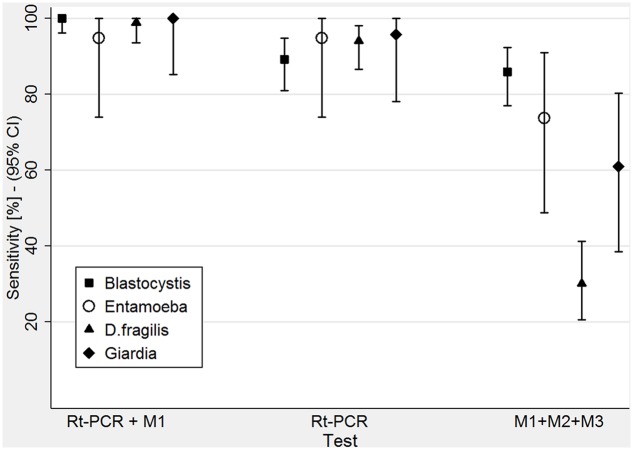
Comparison of the sensitivity of the three tests used: Rt-PCR + M1 (microscopy on one sample), Rt-PCR alone and M1 + M2 + M3 (microscopy on all three samples).

All consecutive patients having provided written informed consent for the use of their biological samples by the CTD, Negrar, Verona, Italy, who were requested a stool microcopy exam and a Rt-PCR for intestinal protozoa between 01.01.2014 and 31.12.2015 (**Figure 1**).

#### Test Methods

##### Stool microscopy

Following the protocol of our laboratory, stool microscopy was performed on three stool samples collected in 10% formalin on consecutive or alternate days. The samples were concentrated according to a modified Ritchie’s method (Buonomini et al., 1956) before the microscopic analysis.

##### DNA extraction and Rt-PCR

Stool specimens were collected as described previously (Formenti et al., 2015) according to the protocol procedure of our laboratory. In detail, 200 mg of stool were stored at -20°C overnight in a solution of 1X PBS with 2% polyvinylpolypyrrolidone (PvPP) (Sigma–Aldrich, Milan, Italy). In each sample, Phocine Herpes Virus type-1 (PhHV-1, kindly provided by Dr. Pas S., Erasmus MC, Department of Virology, Rotterdam) was added to the S.T.A.R. buffer (Roche), serving as an internal control for the isolation and amplification steps. Prior to DNA extraction, all the samples were frozen and then boiled for 10 min at 100°C. The DNA was extracted using the MagnaPure LC.2 instrument (Roche Diagnostic, Monza, Italy), following the protocol “DNA I Blood_Cells High performance II,” using the kit “DNA isolation kit I” (Roche). The DNA was eluted in a final volume of 100 ul.

The Rt-PCR targets are shown in **Table 1**. In brief, amplification reactions for all the Rt-PCR were performed in 25 ul volumes containing PCR buffer (SsoFast master mix, Bio-Rad Laboratories, Milan, Italy), 2.5 ug of BSA (Sigma–Aldrich), 80 nM of each of the PhHV-1 specific primers, and 200 nM of PhHV-1 CY5-BHQ2 labeled probe. Depending on the multiplex performed, we had the following protocol of primers/probes concentration:

**Table 1:** Rt-PCR amplification targets.

Parasite	Gene_target	Reference	Gene_Bank
*Entamoeba histolytica Entamoeba dispar*	SSU rRNA	Verweij et al., 2003a	X64142 Z49256
*Blastocystis* sp.	18S ribosomal RNA gene	Stensvold et al., 2012	AY244621
*Dientamoeba fragilis*	SSU rRNA	Verweij et al., 2007	DQ233450
*Giardia duodenalis*	SSU rRNA	Verweij et al., 2003b	M54878

1. 300 nM of each *Giardia intestinalis* specific primers, 200 nM of *G. intestinalis* CY5.5-BHQ3 labeled probe. 100 nM of each *D. fragilis* specific primers and 100 nM of *D. fragilis* VIC-MGB labeled probe. 300 nM of each *Blastocystis* sp. specific primers and 100 nM of *Blastocystis* sp. FAM-MGB labeled probe.
2. 60 nM of each *E. histolytica/E. dispar* specific primers and 200 nM of *E. histolytica* FAM-MGB labeled probe and *E. dispar* VIC-MGB labeled probe. 200 nM of each *Cryptosporidium* sp. specific primers and 100 nM of *Cryptosporidium* sp. CY5.5-BHQ3 labeled probe.

The Rt-PCR cycle protocol consists of 3 min at 95°C followed by 40 cycles of 15 s at 95°C and 30 s at 60°C, and 30 s at 72°C. The reactions, detection and data analyses were performed with the CFX 96 detection system (Biorad Laboratories) using white plates. Positive and negative controls were included in all the experiments; in detail as positive control we used two pool of positive DNA for the targets included in the multiplex. One had a low Ct (30 < Ct < 36) and the other a high Ct (37 < Ct < 39.9). For all the Rt-PCR analysis, the threshold was set at 200. As a control for Rt-PCR inhibitors and amplification, the exogenous PhHV-1 DNA was amplified with the appropriate primers/probe mix.

### Statistical Analysis

In studies of diagnostic accuracy, the results of one or more tests under evaluation (index tests) are compared with those obtained with the reference standard, both measured in subjects who are suspected of having the condition of interest. In this framework, we considered as the reference standard the extended routine (coproparasitological exam of three samples plus Rt-PCR) while the *index test* was represented by the new proposed, restricted routine (coproparasitological exam of one sample plus Rt-PCR). For the assessment of microscopic accuracy of one single sample, we conventionally considered the first of each series of three samples (Supplementary Information File). We assessed the sensitivity of the index test and its 95% confidence interval [as the specificity of the restricted routine could not logically be lower than that of the extended routine, moreover Rt-PCR specificity was proven to be 100% (Stark et al., 2006; Stensvold et al., 2011) and so is conventionally the specificity of microscopy].

The analysis was performed with STATA vers. 14 (StataCorp, 4905 Lakeway Dr., College Station, TX 77845, United States) and a *p*-value of 5% was considered statistically significant.

## Results

### *Blastocystis* sp.

A total of 277 samples were analyzed by microscopy and Rt-PCR for *Blastocystis* sp.

The new diagnostic approach resulted in 100.0% sensitivity (CI 96.1–100.0%) (**Table 2**). The microscopy-only performed on three samples resulted in 85.9% sensitivity (CI 77.0–92.3%) (**Table 3**). Rt-PCR (**Table 4**) without microscopy resulted in 89.1% sensitivity (CI 80.9–94.7%).

**Table 2:** Primary objective.

	Rt-PCR + M1											
Reference	Blastocystis			Entamoeba *h/d*			*D. fragilis*			*Giardia*	
Rt-PCR + M1 + M2 + M3	+	-	Total	+	-	Total	+	-	Total	+	-	Total
POS	92	0	92	18	1	19	82	1	83	23	0	23
NEG	0	185	185	0	238	238	0	410	410	0	464	464
Total	92	185	277	18	239	257	82	411	493	23	464	487
				
*Sensitivity % (95% CI)*	***100.0 (96.1–100.0)***			***94.7 (74.0–99.9)***			***98.8 (93.5–100.0)***			***100.0 (85.2–100.0)***		

*Reference: Rt-PCR + Microscopy on all the three samples (M1+M2+M3).*

**Table 3:** Secondary objective (I).

	M1+M2+M3											
Reference	*Blastocystis*			*Entamoeba h/d*			*D. fragilis*			*Giardia*	
Rt-PCR + M1 + M2 + M3	+	-	Total	+	-	Total	+	-	Total	+	-	Total
POS	79	13	92	14	5	19	25	58	83	14	9	23
NEG	0	185	185	0	238	238	0	410	410	0	464	464
Total	79	198	277	14	243	257	25	468	493	14	473	487
				
*Sensitivity % (95% CI)*	***85.9 (77.0–92.3)***			***73.7 (48.8–90.9)***			***30.1 (20.5–41.2)***			***60.9 (38.5–80.3)***		

*Reference: Rt-PCR + Microscopy on all the three samples (M1 + M2 + M3).*

**Table 4:** Secondary objective (II).

	Rt-PCR											
Reference	Blastocystis			*Entamoeba h/d*			*D. fragilis*			*Giardia*	
Rt-PCR + M1 + M2 + M3	+	-	Total	+	-	Total	+	-	Total	+	-	Total
POS	82	10	92	18	1	19	78	5	83	22	1	23
NEG	0	185	185	0	238	238	0	410	410	0	464	464
Total	82	195	277	18	239	257	78	415	493	22	465	487
				
*Sensitivity % (95% CI)*	***89.1 (80.9–94.7)***			***94.7 (74.0–99.9)***			***94 (86.5–98.0)***			***95.7 (78.1–99.9)***		

*Reference: Rt-PCR + Microscopy on all the three samples (M1 + M2 + M3).*

### Entamoeba histolytica/dispar

A total of 257 samples were analyzed by microscopy and Rt-PCR for *E. histolytica/dispar*.

The sensitivity of the new diagnostic approach was 94.7% (CI 74.0–99.9%) (**Table 2**). That of microscopy-only performed on three samples was 73.7% (CI 48.8–90.9%) (**Table 3**). Rt-PCR (**Table 4**) without microscopy had a sensitivity of 94.7% (CI 74.0–99.9%).

### Dientamoeba fragilis

A total of 493 samples were analyzed by microscopy and Rt-PCR for *D. fragilis*.

The new diagnostic approach resulted 98.8% sensitive (CI 93.5–100.0%) (**Table 2**). The sensitivity of microscopy-only (three samples) was 30.1% (CI 20.5–41.2%) (**Table 3**), that of Rt-PCR (**Table 4**) without microscopy was 94.0% (CI 86.5–98.0%).

### Giardia intestinalis

A total of 487 samples were analyzed by microscopy and Rt-PCR for *G. intestinalis*.

The sensitivity of the new diagnostic approach was 100.0% (CI 85.2–100.0%) (**Table 2**), that of microscopy-only was 60.9% (CI 38.5–80.3%) (**Table 3**), that of Rt-PCR alone (**Table 4**) resulted 95.7% (CI 78.1–99.9%).

## Discussion

The comparison of microscopy with Rt-PCR for protozoan infection has already been considered by other studies (Stark et al., 2006; ten Hove et al., 2007; Bruijnesteijn van Coppenraet et al., 2009; Verweij, 2014). These studies reported the higher sensitivity of Rt-PCR compared to the microscopy technique. The originality of our work is the assessment of a possible new approach to the routine laboratory approach to parasite diagnosis. Infections by protozoa are still common in Italy, and are very frequent in immigrants and travelers. Helminth ova or larvae are less frequently found in stools, and we did not have a sufficient number of positive samples to do an appropriate analysis. On the other hand, alternative diagnostic tools are available for the most important helminth infections: serology in particular has been shown to be more sensitive than fecal techniques for helminth infections of primary medical impostance such as strongyloidiasis and schistosomiasis, for example (Buonfrate et al., 2015; Beltrame et al., 2017).

**FIGURE 2:**
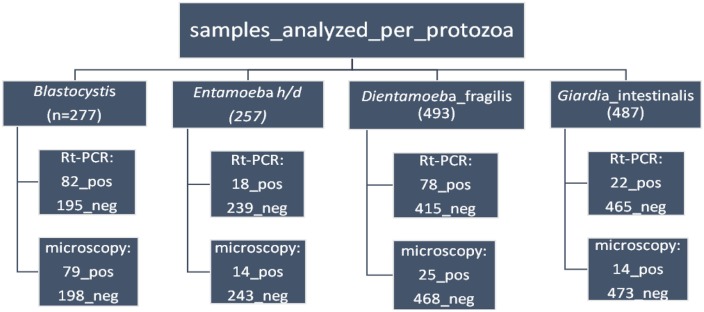
Flowchart of the samples analyzed by Rt-PCR and microscopy.

In this work we retrospectively analyzed and focused on the data of the patients who had been requested a coproparasitological exam on three samples and Rt-PCR for protozoa. The reference standard considered in this study was the extended routine adopted by our laboratory once molecular biology was available, that added a Rt-PCR on one sample to the previous routine (coproparasitological exam on three samples). The “index test” was the proposed, restricted routine (both exams on one single sample). As illustrated in **Table 2**, the new protocol showed an excellent sensitivity for the protozoa analyzed (**Figure 2**), reaching 100% for *Blastocystis* sp. and for *G. intestinalis* and approaching this target for the other two protozoa analyzed, missing just one positive result, respectively, for *E. histolytica*/*dispar* and for *Dientamoeba*.

The sensitivity of the “old” routine coproparasitological exam on three samples without Rt-PCR (**Table 3**) gave different results depending on the target analyzed, but was invariably lower (exceedingly so for *D. fragilis* and *G. intestinalis*) than that of the new, restricted routine. Conversely, Rt-PCR without microscopy showed an excellent sensitivity for all the targets analyzed, although slightly lower (with the notable exception of the target *E. histolytica/dispar*) than that of the new proposed, restricted routine.

## Conclusion

The present study shows that the sensitivity of the new proposed approach for the diagnosis of intestinal protozoa infection remains very high despite the reduction of fecal samples from three to one. Furthermore, it has the advantage of reducing costs and saving time of laboratory personnel. It is also, obviously, a much more practical approach for patients, allowing the sample collection at the moment of the outpatient contact with no need of further visits. It could be argued that Rt-PCR, given the higher sensitivity confirmed by our study, could completely replace microscopy. We disagree for four good reasons: (a) our data show that adding microscopy has the potential of enhancing the sensitivity of the diagnostic approach (although only for *Blastocystis* the difference was statistically significant); (b) microscopy can detect other parasitic infections that may not be included in the Rt-PCR targets requested; (c) residual DNA may persist and be detected by Rt-PCR after parasite clearance, potentially causing false-negative results (Frickmann et al., 2015; Meurs et al., 2017) [others authors obtained conflicting results, showing a fast DNA clearance after treatment (Mejia et al., 2013; van den Bijllaardt et al., 2014)]; (d) last but perhaps most important, good microscopists are becoming a species in danger of extinction and it would be a pity to disperse their precious skills and know-how, not only for protozoa of course, but also for helminths that were not targeted by this study for the reasons explained above.

## Author Contributions

FF conducted the work and wrote the paper; MV performed the statistical analysis; MG contributed to perform the statistical analysis; FP and ZB revised the article; BP performed the molecular analysis; MM, ST, and MD performed the microscopy analysis.

## Conflict of Interest Statement

The authors declare that the research was conducted in the absence of any commercial or financial relationships that could be construed as a potential conflict of interest.
