# Supplementary material for: Molecular Biology Can Change the Classic Laboratory Approach for Intestinal Protozoan Infections
Source: Front Microbiol. 2017 Nov 7;8:2191. doi: 10.3389/fmicb.2017.02191 (PMC5681914; doi:10.3389/fmicb.2017.02191)
Supplement: Supplementary file 1 [file Data_Sheet_1.docx]

**Supplementary_data: comparison between microscopy analysis on the 1**°**, 2**° **and 3**° **sample**

**Blastocystis**

**// Microscopy analysis**

// 1° sample VS 1°+2°+3°

Ris. Mic. | Ris. Mic. M1

M123 | neg pos | Total

-----------+----------------------+----------

neg | 199 0 | 199

| 100.00 0.00 | 100.00

-----------+----------------------+----------

pos | 19 61 | 80

| 23.75 76.25 | 100.00

-----------+----------------------+----------

Total | 218 61 | 279

| 78.14 21.86 | 100.00

// 2° sample VS 1°+2°+3°

Ris. Mic. | Ris. Mic. M2

M123 | neg pos | Total

-----------+----------------------+----------

neg | 199 0 | 199

| 100.00 0.00 | 100.00

-----------+----------------------+----------

pos | 7 73 | 80

| 8.75 91.25 | 100.00

-----------+----------------------+----------

Total | 206 73 | 279

| 73.84 26.16 | 100.00

// 3° sample VS 1°+2°+3°

Ris. Mic. | Ris. Mic. M3

M123 | neg pos | Total

-----------+----------------------+----------

neg | 199 0 | 199

| 100.00 0.00 | 100.00

-----------+----------------------+----------

pos | 12 68 | 80

| 15.00 85.00 | 100.00

-----------+----------------------+----------

Total | 211 68 | 279

| 75.63 24.37 | 100.00

**Entamoeba**

**// Microscopy analysis**

// 1° sample VS 1°+2°+3°

Ris. Mic. | Ris. Mic. M1

M123 | neg pos | Total

-----------+----------------------+----------

neg | 244 0 | 244

| 100.00 0.00 | 100.00

-----------+----------------------+----------

pos | 1 13 | 14

| 7.14 92.86 | 100.00

-----------+----------------------+----------

Total | 245 13 | 258

| 94.96 5.04 | 100.00

// 2° sample VS 1°+2°+3°

Ris. Mic. | Ris. Mic. M2

M123 | neg pos | Total

-----------+----------------------+----------

neg | 244 0 | 244

| 100.00 0.00 | 100.00

-----------+----------------------+----------

pos | 0 14 | 14

| 0.00 100.00 | 100.00

-----------+----------------------+----------

Total | 244 14 | 258

| 94.57 5.43 | 100.00

3° sample VS 1°+2°+3°

Ris. Mic. | Ris. Mic. M3

M123 | neg pos | Total

-----------+----------------------+----------

neg | 244 0 | 244

| 100.00 0.00 | 100.00

-----------+----------------------+----------

pos | 1 13 | 14

| 7.14 92.86 | 100.00

-----------+----------------------+----------

Total | 245 13 | 258

| 94.96 5.04 | 100.00

**Dientamoeba**

**// Microscopy analysis**

// 1° sample VS 1°+2°+3°

Ris. Mic. | Ris. Mic. M1

M123 | neg pos | Total

-----------+----------------------+----------

neg | 470 0 | 470

| 100.00 0.00 | 100.00

-----------+----------------------+----------

pos | 12 13 | 25

| 48.00 52.00 | 100.00

-----------+----------------------+----------

Total | 482 13 | 495

| 97.37 2.63 | 100.00

// 2° sample VS 1°+2°+3°

Ris. Mic. | Ris. Mic. M2

M123 | neg pos | Total

-----------+----------------------+----------

neg | 470 0 | 470

| 100.00 0.00 | 100.00

-----------+----------------------+----------

pos | 12 13 | 25

| 48.00 52.00 | 100.00

-----------+----------------------+----------

Total | 482 13 | 495

| 97.37 2.63 | 100.00

// 3° sample VS 1°+2°+3°

Ris. Mic. | Ris. Mic. M3

M123 | neg pos | Total

-----------+----------------------+----------

neg | 470 0 | 470

| 100.00 0.00 | 100.00

-----------+----------------------+----------

pos | 16 9 | 25

| 64.00 36.00 | 100.00

-----------+----------------------+----------

Total | 486 9 | 495

| 98.18 1.82 | 100.00

**Giardia**

**// Microscopy analysis**

// 1° sample VS 1°+2°+3°

Ris. Mic. | Ris. Mic. M1

M123 | neg pos | Total

-----------+----------------------+----------

neg | 475 0 | 475

| 100.00 0.00 | 100.00

-----------+----------------------+----------

pos | 2 12 | 14

| 14.29 85.71 | 100.00

-----------+----------------------+----------

Total | 477 12 | 489

| 97.55 2.45 | 100.00

// 2° sample VS 1°+2°+3°

Ris. Mic. | Ris. Mic. M2

M123 | neg pos | Total

-----------+----------------------+----------

neg | 475 0 | 475

| 100.00 0.00 | 100.00

-----------+----------------------+----------

pos | 2 12 | 14

| 14.29 85.71 | 100.00

-----------+----------------------+----------

Total | 477 12 | 489

| 97.55 2.45 | 100.00

// 3° sample VS 1°+2°+3°

Ris. Mic. | Ris. Mic. M3

M123 | neg pos | Total

-----------+----------------------+----------

neg | 475 0 | 475

| 100.00 0.00 | 100.00

-----------+----------------------+----------

pos | 1 13 | 14

| 7.14 92.86 | 100.00

-----------+----------------------+----------

Total | 476 13 | 489

| 97.34 2.66 | 100.00
